# Metformin Attenuates Slow-to-Fast Fiber Shift and Proteolysis Markers Increase in Rat Soleus after 7 Days of Rat Hindlimb Unloading

**DOI:** 10.3390/ijms24010503

**Published:** 2022-12-28

**Authors:** Kristina A. Sharlo, Irina D. Lvova, Svetlana P. Belova, Ksenia A. Zaripova, Boris S. Shenkman, Tatiana L. Nemirovskaya

**Affiliations:** Institute of Biomedical Problems, RAS, 123007 Moscow, Russia

**Keywords:** hindlimb unloading, atrophy, AMPK, myosin, proteolysis

## Abstract

Muscle unloading leads to signaling alterations that cause muscle atrophy and weakness. The cellular energy sensor AMPK can regulate myofiber-type shift, calcium-dependent signaling and ubiquitin-proteasome system markers. We hypothesized that the prevention of p-AMPK downregulation during the first week of muscle unloading would impede atrophy development and the slow-to-fast shift of soleus muscle fibers, and the aim of the study was to test this hypothesis. Thirty-two male Wistar rats were randomly assigned to four groups: placebo control (C), control rats treated with metformin (C + M), 7 days of hindlimb suspension (HS) + placebo (7HS), and 7 days of HS + metformin administration (7HS + M). In the soleus of the 7HS rats, we detected a slow-to-fast fiber-type shift as well as a significant downregulation of MEF-2D and p300 in the nuclei. In the 7HS group, we also found decreases in p-ACC (AMPK target) protein level and in the expression of E3 ubiquitin ligases and p-CaMK II protein level vs. the C group. The 7-day metformin treatment for soleus muscle unloading (1) prevented slow-to-fast fiber-type shift; (2) counteracted changes in the p-ACC protein level; (3) hindered changes in the nuclear protein level of the slow myosin expression activators MEF-2D and p300, but did not affect NFATc1 signaling; and (4) attenuated the unloading-induced upregulation of MuRF-1, atrogin-1, ubiquitin and myostatin mRNA expression, but did not prevent soleus muscle atrophy. Thus, metformin treatment during muscle disuse could be useful to prevent the decrease in the percentage of slow-type fatigue-resistant muscle fibers.

## 1. Introduction

Skeletal muscle unloading leads to both structural and functional negative changes. These changes include muscle fiber atrophy, slow-to-fast fiber-type shift and the lowering of muscle oxidative capacity [1]. Slow-to-fast fiber-type transformation and myofiber atrophy result in altered muscle fatigue resistance and force that can negatively affect muscle performance [2,3]. Muscle unloading occurs during space flight missions, under bed rest conditions in chronically ill patients, and in animal models of unloading (rodent hindlimb suspension).

AMPK (AMP-activated protein kinase) is an energy sensor kinase that is regulated by the AMP/ATP ratio and controls numerous muscle characteristics, such as muscle fiber type, proteolysis rate and muscle oxidative capacity [4,5]. After 1- and 3-day hindlimb suspension, phosphorylated AMPK (p-AMPK) is significantly downregulated and ATP is accumulated in rat soleus muscle [6,7,8]. After 14 days of rat hindlimb suspension, as well as after 14-day denervation, ATP level is significantly decreased [9] and p-AMPK protein level at this time point exceeds control values [7]. Seven-day rat hindlimb suspension is a “borderline” time point when ATP content and p-AMPK protein level may be either decreased or be equivalent to control values [7,10]. In contrast to the 7-day time point, after 3 days of rat hindlimb unloading, the downregulation of slow-type myosin heavy chain (MyHC I) and the changes in slow-to-fast fiber-type ratio usually cannot be identified [11,12,13]. In this study, a 7-day time point of rat hindlimb suspension was chosen in order to detect the unloading-induced alterations in the number of slow-type and fast-type myofibers. A 3-day hindlimb suspension time point was also added to see if metformin treatment prevents p-AMPK downregulation at this stage.

The unloading-induced lack of p-AMPK in the soleus muscle is accompanied by the rapid accumulation of calcium ions in the sarcoplasm [14,15], as well as by dramatic ATP accumulation during 1–3 days of muscle unloading [16]. Previously, it was found that AMPK inactivation under rat hindlimb suspension leads to alterations in soleus muscle resting membrane potential that promote the opening of voltage-sensitive DHPR channels and sarcoplasmic calcium accumulation [17,18]. The opening of DHPR and calcium accumulation in the sarcoplasm may lead to the destabilization of ryanodine receptors and calcium leakage from SR, causing a self-sustaining cycle [19,20]. Another mechanism of AMPK-dependent calcium signaling regulation includes the phosphorylation of SERCA inhibitor phospholamban, which leads to disrupted phospholamban-SERCA interaction and activated SR calcium uptake. The inactivation of this mechanism due to unloading-induced p-AMPK decline may also contribute to excessive calcium accumulation [21]. Unloading-induced calcium accumulation in skeletal muscle can lead to proteolytic pathway activation and muscle atrophy (defined as a decrease in muscle mass and fiber CSA) [16,22,23,24]. Thus, based on these data, we suppose that preventing the lowering of p-AMPK protein level during 7 days of rat hindlimb suspension would counteract calcium signaling changes and impede the upregulation of proteolytic markers in soleus muscle.

AMPK regulates a number of calcium signaling pathways. For example, a crosstalk between CaMKKβ and AMPK, as well as between CaMK II and AMPK, has been detected [25,26]. CaMK II, in turn, regulates calcium-dependent transcriptional repressors and activators, including those controlling muscle fiber-type regulation (such as HDAC4 [27] and NFAT (via calcineurin) [28]). 

The slow-to-fast fiber-type shift in skeletal muscles can be regulated via calcineurin/NFATc1 and HDAC4/MEF-2 [29]. Both MEF-2 and NFATc1 bind to the promoter of slow-type myosin (*myh 7*) gene and enhance its transcription [30]. AMPK regulates each of these pathways via different signaling mechanisms [31,32,33]. 

The calcineurin/NFATc1 pathway is activated in myofibers in response to calcium and NO signals [29,34]. At the same time, during muscle unloading, NFATc1 is inactivated despite the unloading-induced sarcoplasmic calcium ion accumulation [13]. Previously, we have shown that the unloading-induced activation of CaMK II-dependent MAP-kinase p38 [35] leads to NFATc1-signalling inactivation [36]; thus, it can be suggested that during muscle unloading, the Ca^2+^/CaMK II/p38 pathway can suppress NFATc1. Reduced p-AMPK protein levels induced by soleus muscle unloading may contribute to sarcoplasmic Ca^2+^ accumulation [17,18] leading to CaMK II autophosphorylation and activation [37]. CaMK II, in turn, can activate MAP-kinase 38 in skeletal muscles [38]. We hypothesized that metformin can downregulate CaMKII/p38 by preventing p-AMPK protein level decline. P38 inactivation could, in turn, lead to NFATc1 activation and slow myosin expression. 

Another myosin-regulating pathway, HDAC4/MEF-2, could be directly controlled via AMPK. Deacetylating and inactivating the MEF-2 transcription factor by HDACs represses slow myosin gene transcription, while the acetylation of MEF-2 leads to the opposite effect [30,39]. AMPK can phosphorylate histone deacetylase 4, leading to the export of HDAC4 from the myonuclei and its inability to deacetylate MEF-2 [40]. AMPK activity can also control MEF-2 nuclear accumulation in C2C12 myotubes [32]. Earlier, it was shown that MEF-2 nuclear protein level declines after both rat hindlimb suspension and space flight [10,41], and this event is accompanied by slow-to-fast fiber-type transformation; thus, it cannot be excluded that unloading-induced slow-to-fast fiber-type transformation may be, at least in part, mediated by the elimination of MEF-2 from the nucleus. At the same time, the causes of reduced MEF-2 nuclear protein levels during muscle unloading remain unknown.

We have examined whether metformin, a well-known AMPK activator, affects slow-to-fast fiber-type transformation, myosin-regulating transcription factors MEF-2 or NFATc1, nuclear protein level, or ubiquitin–proteasome marker upregulation during 7 days of rat hindlimb suspension.

## 2. Results

### 2.1. ATP Content and p-ACC/p-AMPK Protein Levels after 3 Days and 7 Days of Rat Hindlimb Unloading

Before the 7-day experiment, we conducted a preliminary experiment to test if metformin prevents ATP accumulation and AMPK inactivation at the early stage of unloading (3 days).

**Figure 1:**
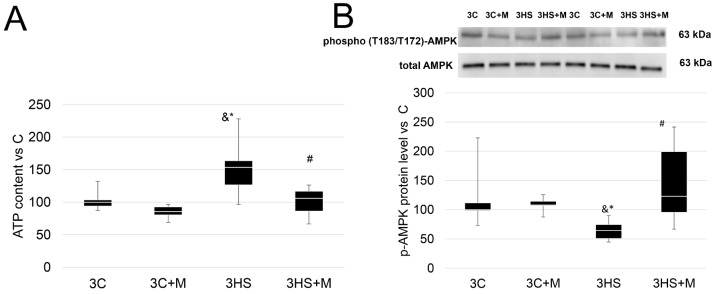
ATP content (**A**) and p-AMPKα1/2 (T183/T172) protein level (**B**) in rat soleus muscle of the control group (3C), the control group with metformin (3C+M), the 3-day hindlimb-suspended group (3HS), and the 3-day hindlimb-suspended group with metformin (3HS+M). Data are shown as % of the control group, and median of the C group has been arbitrarily set to 100%. *—significant difference from the control group. &—significant difference from 3C+M group. #—significant difference from 3HS group (*p* < 0.05). Box plots show 25–75 percentiles and median values and the whiskers represent the minimum and the maximum; n = 8/group.

After 3 days of rat hindlimb suspension, the content of ATP was higher by 150% compared to 3C and 3C+M groups. In the 3HS+M group (with metformin administration), this effect was completely prevented (Figure 1A). The protein level of p-AMPK was significantly lower in group 3HS compared with 3C and 3C+M, while in the 3HS+M group, the protein level was significantly higher than in the 3HS group, and did not differ from the 3C and 3C+M groups (Figure 1B).

**Figure 2:**
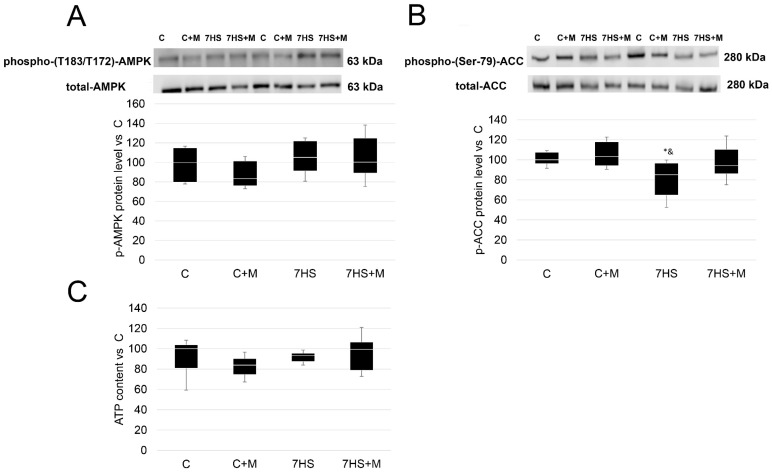
Western blot analysis of p-AMPKα1/2(T183/T172) protein level (**A**), p-ACC (Ser 79) protein level (**B**), and ATP content (**C**) in rat soleus muscle in the control group (C), control group with metformin (C+M), 7-day hindlimb-suspended group (7HS), and 7-day hindlimb-suspended group with metformin (7HS+M). Data are shown as % of the control group, and median of the C group has been arbitrarily set to 100%. *—significant difference from the control group. &—significant difference from C+M group (*p* < 0.05). Box plots show 25–75 percentiles and median values, and the whiskers represent the minimum and the maximum; n = 8/group.

After 7 days of HS p-AMPK (T183/172), the protein level did not differ among the experimental groups. At the same time, in the 7HS group, the protein level of the AMPK target, p-ACC (Ser 79), was lower compared with the C and C+M groups. In the 7HS+M group, the protein level of p-ACC did not differ from the C or C+M rats. The C and C+M rats’ results did not differ from one another (Figure 2).

### 2.2. Influence of Metformin Administration on MyHC Expression and Fiber-Type Ratio

**Figure 3:**
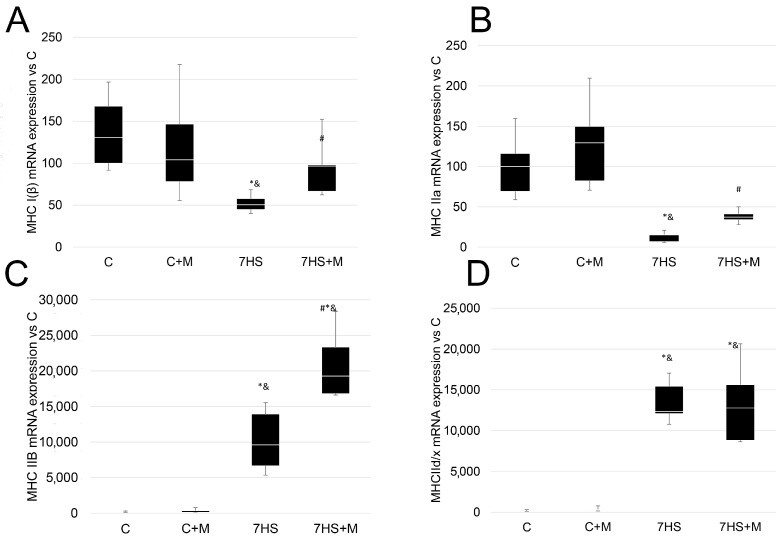
Expression of MyHC I(β) (**A**), MyHC IIa (**B**), MyHC IIb (**C**), and MyHC IId/x (**D**) mRNAs in rat soleus muscle in the control group (C), control group with metformin (C+M), 7-day hindlimb-suspended group (7HS), and 7-day hindlimb-suspended group with metformin (7HS+M). Data are shown as % of the control group, and median of the C group has been arbitrarily set to 100%. *—significant difference from the control group. &—significant difference from C+M group. #—significant difference from 7HS group (*p* < 0.05). Box plots show 25–75 percentiles and median values and the whiskers represent the minimum and the maximum; n = 8/group.

The expression of all myosin isoforms of mRNA did not differ between the C and C+M rats. In the 7HS group, the expression of the “slow” isoforms of myosin heavy-chain (MyHC I) mRNA in soleus muscle was significantly lower by 50% compared with the C and C + M groups. At the same time, in the 7HSM group, the expression of MyHC I mRNA was significantly higher than in the 7HS group, and had no significant differences from C and C+M groups (Figure 3A). 

The expression of MyHC IIa mRNA in the soleus muscles of the 7HS rats was also significantly lower compared with the C and C + M rats: it was 15% of the C group level. In the 7HS+M rats, the expression of MyHC IIa mRNA was significantly lower than in the C and C + M groups; however, it was significantly higher compared with the 7HS rats (Figure 3B). 

In the 7HS group, the mRNA of “fast” myosin isoforms IIB and IId/x were upregulated by 12.3 times and 12.7 times compared with group C, respectively. Additionally, in the 7HS group, the expressions of both “fast” myosin isoforms IIB and IId/x were significantly higher than in the C+M group. In the 7HS+M group, the expression of mRNA of myosin isoform IId/x did not differ from the 7HS group, and it was significantly higher in comparison with the C and C+M groups. The expression of “fast” myosin IIB in the 7HS+M group was significantly higher than in all other experimental groups (Figure 3C,D).

**Figure 4:**
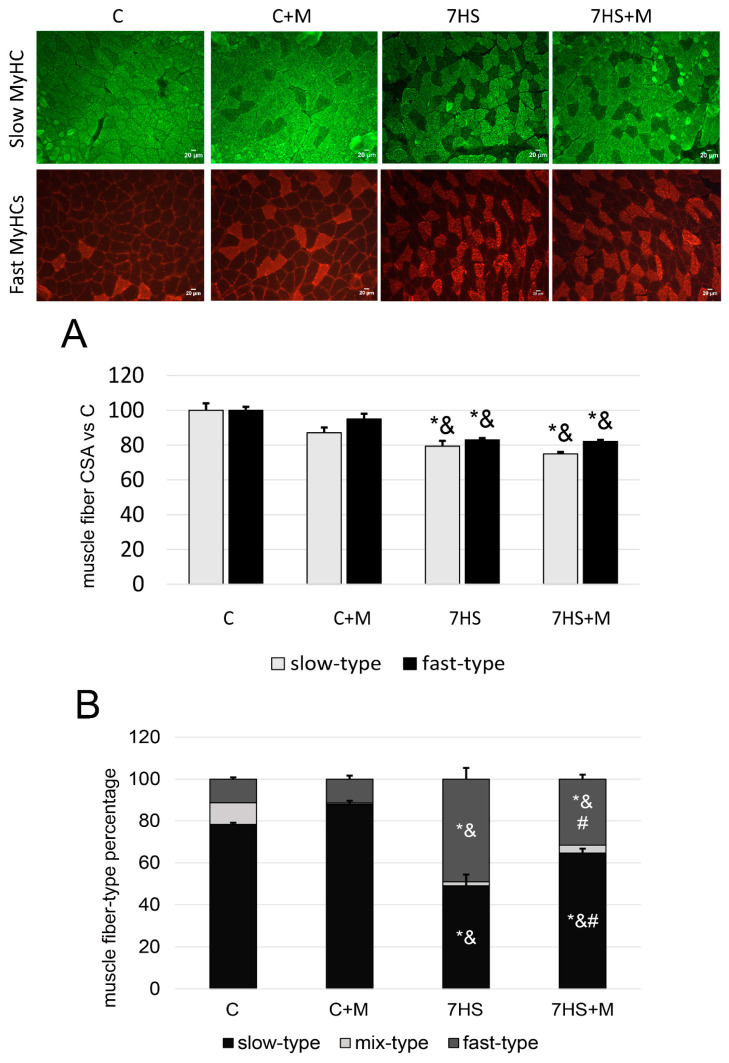
Immunohistochemical analysis of slow-type and fast-type myofiber relative percentage vs. control, arbitrarily set at 100% (**A**), and fibers’ CSA (**B**) in the control group (C), control group with metformin (C+M), 7-day hindlimb-suspended group (7HS), and 7-day hindlimb-suspended group with metformin (7HS+M). Data are shown as % of the control group, and mean value of the C group has been arbitrarily set to 100%. *—significant difference from the control group. &—significant difference from C+M group. #—significant difference from 7HS group (*p* < 0.05). Graphs represent mean value ± SEM; n = 8/group.

The percentages of slow-type and fast-type fibers did not significantly differ between the C and C+M groups, or between the slow-type and fast-type fibers’ cross-sectional areas (CSAs). In the 7HS group, the percentage of slow-type fibers was less and the percentage of fast-type fibers was greater compared with the C and C+M groups. Both slow-type and fast-type fibers’ CSAs in the 7HS group were significantly smaller than in C and C+M groups (Figure 4B). In the 7HS+M group, the percent of slow-type fibers was significantly higher than in 7HS group, although it was significantly lower than in the C and C+M groups. At the same time, in the 7HS+M group, the percent of fast-type fibers was significantly lower than in the 7HS group, although it was significantly greater compared with the C and C+M groups. In the 7HS+M group, the slow-type and fast-type fibers’ CSAs did not significantly differ from 7HS group, and they were both smaller than in the C and C+M groups.

**Figure 5:**
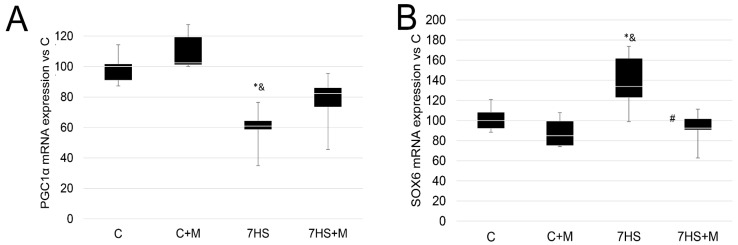
RT-PCR analysis of PGC1alpha mRNA expression (**A**) and SOX6 mRNA expression (**B**) in the control group (C), control group with metformin (C+M), 7-day hindlimb-suspended group (7HS), and 7-day hindlimb-suspended group with metformin (7HS+M). Data are shown as % of the control group and median of the C group has been arbitrarily set to 100%. *—significant difference from the control group. &—significant difference from C+M group. #—significant difference from 7HS group (*p* < 0.05). Box plots show 25–75 percentiles and median values and the whiskers represent the minimum and the maximum; n = 8/group.

The mRNA expression of the key marker of mitochondrial biogenesis, PGC1α, was significantly less in the 7HS group compared with the C and C+M groups, and amounted to 60% of the control group, while in the 7HS+M group there were no significant differences from either the C or C+M groups, but there was a significant difference from the 7HS group. The mRNA expression of the slow myosin transcriptional repressor SOX6 was significantly higher in the 7HS group by 40% compared with the C and C+M groups, while the C and C+M groups had no significant differences from one another. In the 7HS+M group, the expression of SOX6 mRNA was significantly lower than in the 7HS group and did not differ significantly from the C and CM groups (Figure 5).

### 2.3. Effect of Metformin Administration on MEF-2D and HAT p300 Nuclear Protein Level S

**Figure 6:**
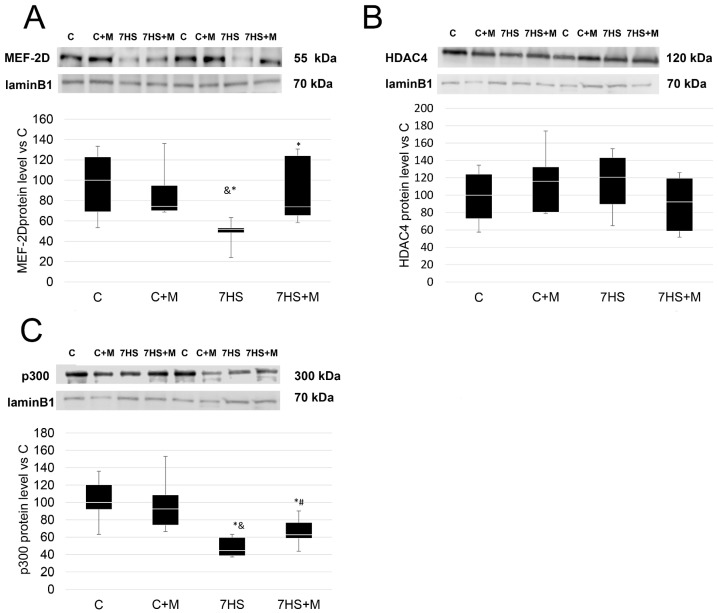
Western blot of MEF-2D (**A**), HDAC4 (**B**) and HAT p300 (**C**) nuclear protein levels in control group (C), control group with metformin (C+M), 7-day hindlimb-suspended group (7HS), and 7-day hindlimb-suspended group with metformin (7HS+M). Data are shown as % of the control group and median of the C group has been arbitrarily set to 100%. *—significant difference from the control group. &—significant difference from C+M group. #—significant difference from 7HS group (*p* < 0.05). Box plots show 25–75 percentiles and median values and the whiskers represent the minimum and the maximum; n = 8/group.

The protein level of the transcription factor MEF-2D in the nuclear fraction of the soleus muscles of the 7HS rats was significantly lower by 80% compared with the C and C + M groups (Figure 6). At the same time, in the 7HS+M group, the protein level of MEF-2D in the nuclear fraction was twice as high as in the 7HS group, and significantly differed from both 7HS and C groups. The protein level of HDAC4 in the nuclear fraction did not differ between the experimental groups. The nuclear protein level of HAT p300 in the 7HS group was significantly lower in comparison with the C and C+M groups, while in the 7HS+M group the protein level of p300 in the nuclear fraction was significantly higher than in the 7HS group, although it was still significantly lower than in the control group. Neither p300 nor MEF-2D nuclear protein levels differed between the C and C+M groups.

### 2.4. Influence of Metformin Administration on Calcineurin/NFAT Pathway upon Hindlimb Suspension

**Figure 7:**
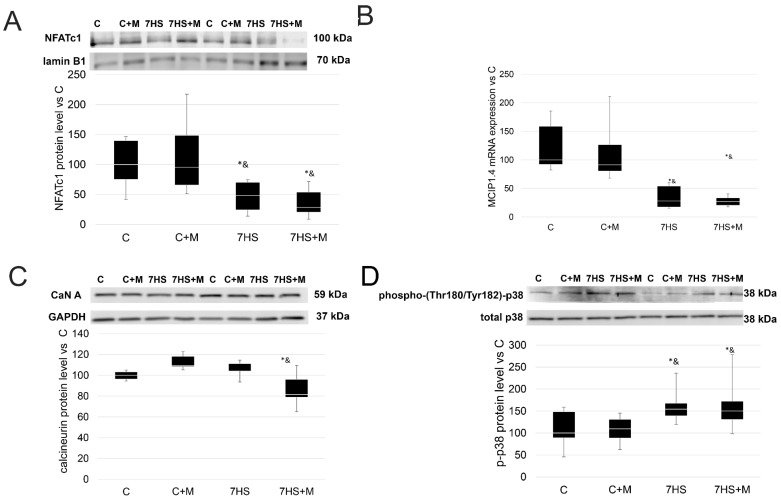
Western blot of nuclear NFATc1 (**A**) and RT-PCR of MCIP1.4 (**B**), and Western blot of calcineurin protein level (**C**) and p-(Thr180/Tyr182) p38 (**D**) in control group (C), control group with metformin (C+M), 7-day hindlimb-suspended group (7HS), and 7-day hindlimb-suspended group with metformin (7HS+M). Data are shown as % of the control group and median of the C group has been arbitrarily set to 100%. *—significant difference from the control group. &—significant difference from C+M group (*p* < 0.05). Box plots show 25–75 percentiles and median values and the whiskers represent the minimum and the maximum; n = 8/group.

In addition to the MEF-2/p300 pathway, calcineurin/NFAT can also take part in myosin gene expression regulation. The expression of MCIP1.4 mRNA (which is an NFATc1 activity marker [42,43]) was significantly less in both the 7HS and 7HS+M groups in comparison with the C and C+M groups, while the C and C+M groups did not differ from each other (Figure 7) The protein level of NFATc1 in the nuclear fraction of the soleus muscles was significantly lower than the C and C+M groups in the 7HS and 7HS+M groups by 80%, while the 7HS and 7HS+M groups did not differ from each other, nor did the C and C+M groups. The protein level of calcineurin A in the total protein fraction of the soleus muscles was significantly lower in the 7HS+M group compared with the C, C+M and 7HS groups, while there were no significant differences among the other groups. Thus, hindlimb unloading led to the inactivation of NFAT-dependent gene expression and NFAT nuclear protein level, and metformin did not prevent this effect. The protein level of Thr180/Tyr182-phosphorylated p38 MAP-kinase did not differ between the C and C+M groups, and it was upregulated by 50% in both the 7HS and 7HS+M groups compared with the C and C+M groups.

### 2.5. Metformin Affects Calcium-Dependent Signaling Markers

**Figure 8:**
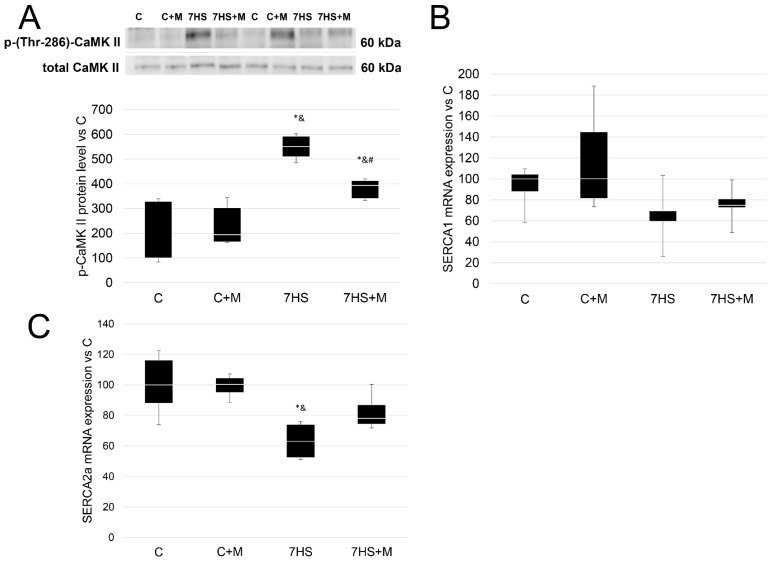
Western blot of p-(Thr286)-CaMK II (beta)/total CaMK II (beta) protein level (**A**), and RT-PCR of SERCA1 (**B**) and SERCA2A mRNA expression in control group (**C**), control group with metformin (C+M), 7-day hindlimb-suspended group (7HS), and 7-day hindlimb-suspended group with metformin (7HS+M). Data are shown as % of the control group and median of the C group has been arbitrarily set to 100%. *—significant difference from the control group. &—significant difference from C+M group. #—significant difference from 7HS group (*p* < 0.05). Box plots show 25–75 percentiles and median values and the whiskers represent the minimum and the maximum; n = 8/group.

All the described parameters did not differ between the C and C+M groups. The protein level of p-(Thr286)-CaMK II (beta) was 5.5-fold higher in the 7HS group compared with the C and C+M groups. In the 7HS+M group, the protein level of p-(Thr286)-CaMK II (beta) was significantly higher than in the C and C+M groups, but also was significantly lower than in the 7HS group. SERCA1 mRNA expression did not differ among the experimental groups. SERCA2a mRNA expression was lower in the 7HS group compared with the C and C+M groups, and in the 7HS+M group this effect was partially prevented so that the 7HS+M group did not differ from any other experimental group (Figure 8).

### 2.6. Influence of Metformin Administration on Ubiquitin–Proteasome Signaling Markers

No analyzed proteolytic markers differed between the C and C + M groups. After 7 days of hindlimb suspension, the mRNA expression of atrogin-1 was two-fold higher compared with the C and C+M groups. In the 7HS+M group, the expression of atrogin-1 mRNA was significantly lower than in the 7HS group and significantly higher than in the C+M group (but did not differ from the C group). The expression of MuRF-1 was significantly greater in the 7HS group compared with the C and C+M groups, and this effect was partially prevented in the 7HS+M group, so this group did not differ significantly from any other experimental group.

The expression of ubiquitin mRNA was significantly higher in the 7HS group compared with the C and C+M groups, while the 7HS+M group did not differ from any experimental group. The expression of myogenin did not differ among the C, C+M and 7HS groups, but it was significantly higher in the 7HS+M group compared with the C group. The expression of myostatin mRNA in the 7HS group was greater by more than three-fold compared with the C and C+M groups, while it was significantly lower in the 7HS+M group than in the 7HS group, and did not differ from the C or C+M groups.

## 3. Discussion

Metformin is widely used as an activator of AMPK, although the detailed mechanisms of this activation remain elusive and include both AMP/ATP-dependent and AMP/ATP-independent mechanisms [44,45]. In the current study, it was found that metformin successfully prevents unloading-induced p-ACC drop, but does not affect p-AMPK or ATP content after 7 days of rat hindlimb unloading. However, it is worth noting that metformin successfully prevented the downregulation of p-AMPK protein level as well as ATP accumulation after 3 days of rat hindlimb unloading (Figure 1A,B). These data are in line with the previous findings obtained at this time point [16,46], which are in contrast to the 7-day time point, when ATP content and p-AMPK protein level may be either slightly lower or equivalent to the control [9,47]. Thus, metformin administration during 3 days of soleus muscle unloading prevented a decline in p-AMPK protein level, and its effects lasted until at least 7 days of rat hindlimb suspension.

At the 7-day time point of rat hindlimb suspension, in contrast to the early stages of unloading, there were detected unloading-induced changes in the number of slow-type and fast-type myofibers, so this time point was chosen to analyze the effect of metformin on fiber-type shift [11,12,13]. At the same time, metformin administration prevented a drop in slow myosin mRNA expression (Figure 3A), but did not prevent the accumulation of fast myosin isoforms (Figure 3B–D). However, it seems that the prevention of slow-type myosin mRNA downregulation is sufficient to partially prevent slow-to-fast fiber-type transformation (Figure 4).

The observed effects of rat hindlimb suspension on soleus muscle myosin expression patterns and slow-to-fast fiber-type transformation correspond to previous data [47,48,49,50,51]. At the same time, this study is the first to show that metformin prevents slow-to-fast fiber-type transformation during a muscle unloading model (Figure 4). The beneficial effect of metformin on the percentage of slow-type muscle fibers was shown previously on obese mice, which agrees well with our study, but in the cited article the potential mechanisms of this effect were not studied [52]. Metformin administration also prevented the unloading-induced PGC1alpha mRNA decrease (Figure 5A). This result is in a good agreement with the earlier reported effects of metformin on skeletal muscle [53]. Previously, it was shown that PGC1alpha can upregulate slow-type and II a myosin expression [54,55], so the metformin-dependent prevention of PGC1alpha mRNA decrease could contribute to the rescue of slow, oxidative myofibers during muscle unloading. PGC1alpha in skeletal muscle was shown to activate mitochondrial biogenesis and muscle oxidative capacity [56]. However, in the study by Wessels et al., metformin worsened muscle oxidative capacity in transgenic healthy and diabetic rats’ tibialis anterior muscles [57], while in the other study, metformin prevented cardiotoxin-induced muscle atrophy and enhanced myofibers’ oxidative metabolism [58]. In this study, we did not test muscle oxidative capacity, so the effect of metformin on the unloaded soleus muscle’s oxidative potential is still unclear.

The increased expression of SOX6, a slow-type gene repressor, was observed at the early stage after 7 days of suspension vs. control in the soleus muscles of rats (Figure 5B); however, the regulatory mechanisms that determine the level of SOX6 expression under muscle unloading are unknown [47]. The metformin-induced inhibition of SOX6 expression is detected for the first time, and this effect could also contribute to the metformin impact on the percentage of slow-type myofibers and slow myosin expression.

Slow myosin expression in skeletal muscles is regulated by two parallel pathways: HDAC4/MEF and NFATc1 [29]. It was previously indicated that, among MEF-2 transcription factors, MEF-2C and MEF-2D activate slow myosin expression [30,41,59,60]; however, MEF-2C nuclear protein level does not change during rat hindlimb unloading, in contrast to MEF-2D [61]. MEF-2D-HDAC4 interaction leads to MEF-2D deacetylation and inactivation [39,62], while P300 acetylates and activates MEF-2 [63]. In this study, it is found that metformin administration partially prevents MEF-2D nuclear protein level decline and fully prevents the decrease in MEF-2D coactivator p300 protein level in myonuclei, but it does not affect HDAC4 nuclear protein level (Figure 6A–C). The observed effect of 7-day hindlimb unloading on MEF-2D and p300 nuclear protein level corresponds to previous findings [64]. MEF-2 nuclear protein level was previously demonstrated to be controlled via AMPK in myotubes [32], so it is possible that metformin administration leads to nuclear MEF-2D accumulation due to AMPK activation. The effect of metformin on p300 nuclear accumulation corresponds to previous findings on the AMPK-dependent activation of p300-mediated acetylation in myonuclei [65]. Thus, the increase in p300 nuclear protein levels accompanied by increased MEF-2D protein levels could result in MEF-2D acetylation and transcriptional activation, leading to a slow-type myofiber phenotype. At the same time, AMPK was shown to import HDAC4 from the myonuclei [7], but such an effect was not observed in the present study. The effect of metformin on p-CaMK II protein level may be caused by the prevention of unloading-induced resting sarcoplasmic calcium ion accumulation. It could be mediated via SERCA2a mRNA expression decrease prevention, or by preventing ATP accumulation at the earlier stage of unloading, which could trigger calcium ion accumulation in slow-type myofibers [66].

We suggested that metformin could prevent NFAT inactivation via CaMK II beta/MAP kinase p38 downregulation. Previously, it was shown that unloading-induced MAP-kinase p38 activation inhibits NFATc1 [36]. However, metformin did not impede NFAT inactivation or p-p38 kinase protein level increase, although it successfully mitigated p-CaMK II protein level upregulation (Figure 7 and Figure 8). Thus, we can conclude that the downregulation of p-CaMK II beta protein level does not lead to the prevention of unloading-induced p38 activation, despite CAMK II beta being an upstream regulator of MAP kinase p38 [38].

It should be noticed that metformin did not affect NFATc1-signalling pathway, but despite that, it still managed to attenuate the unloading-induced slow-to-fast fiber-type shift in soleus muscle. Thus, the simultaneous activation of both NFATc1 and MEF-2 is not indispensable to counteract slow-to-fast fiber-type transformation during muscle unloading. This finding supports previous results indicating that two parallel signaling pathways regulate slow myosin expression [29].

**Figure 9:**
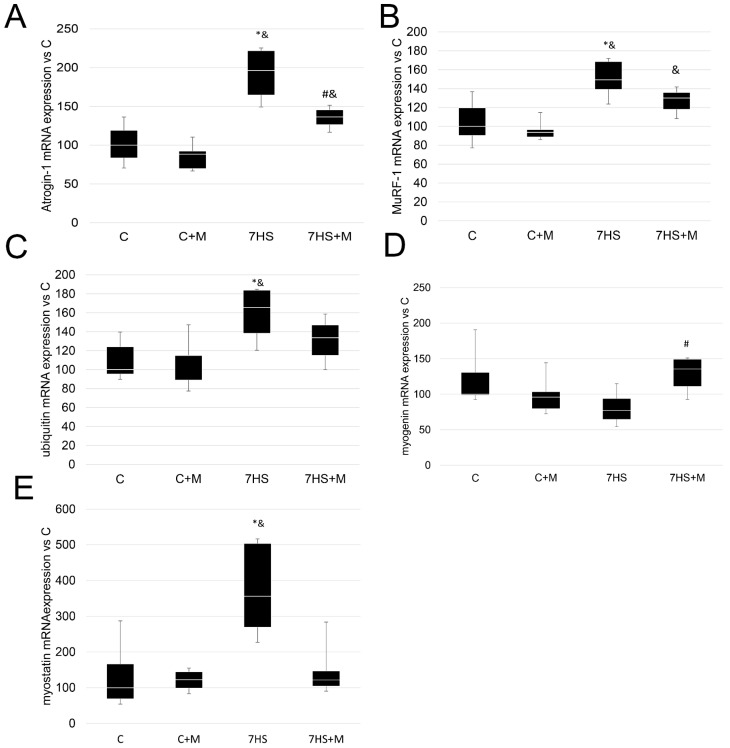
RT-PCR analysis of mRNA expression of atrogin-1 (**A**), MuRF-1 (**B**) ubiquitin (**C**), myogenin (**D**) and myostatin (**E**) in control group (C), control group with metformin (C+M), 7-day hindlimb-suspended group (7HS), and 7-day hindlimb-suspended group with metformin (7HS+M). Data are shown as % of the control group and median of the C group has been arbitrarily set to 100%. *—significant difference from the control group. &—significant difference from C+M group. #—significant difference from 7HS group (*p* < 0.05). Box plots show 25–75 percentiles and median values and the whiskers represent the minimum and the maximum; n = 8/group.

Metformin administration successfully attenuated the upregulation of MuRF-1, atrogin-1, ubiquitin and myostatin under muscle unloading (Figure 9). MuRF-1 and atrogin-1 are key components of the ubiquitin–proteasome system in skeletal muscle [67], and myostatin is a negative regulator of skeletal muscle mass, which can activate MuRF-1 and atrogin-1 expression [68]. Metformin also mitigated myogenin upregulation under 7-day HS. The literature data show that metformin either downregulates proteasome signaling markers or has no effect on skeletal muscle [69,70]. Metformin also upregulates myogenin in obese rodents’ offspring [70]. These results are in a good agreement with our findings. However, in C2C12 cells, metformin was shown to upregulate MuRF-1, myostatin and atrogin-1 expression [71,72]. It also increased myogenin expression in mice; at the same time, the effect of metformin on myostatin in C2C12 myotubes was reversed under AMPKα2 blocking [73]. Based on the literature data and our data, we can suggest that the effects of metformin on the proteolytic system critically depend on the model of muscle atrophy and the state of signaling pathways in the muscle.

It should be also mentioned that, in our study, we observed neither atrophy prevention nor deterioration by metformin, despite the metformin-induced prevention of proteolytic marker upregulation. 

Summing up, we can conclude that the 7-day metformin treatment for soleus muscle unloading (1) prevented a decrease in AMPK target p-ACC protein level; (2) prevented slow-to-fast fiber-type shift and slow myosin mRNA expression downregulation; (3) blocked decreases in nuclear protein levels of slow myosin expression activators MEF-2D and p300, but did not affect NFATc1 signaling; and (4) hindered the unloading-induced upregulation of MuRF-1, atrogin-1, ubiquitin and myostatin mRNA expression, but did not affect muscle atrophy.

## 4. Materials and Methods

### 4.1. Animal Experiments

Gravitational unloading was simulated by using a standard hindlimb suspension (HS) model. A strip of adhesive tape was applied to the animal’s tail, which was suspended by passing the tape through a swivel that was attached to a metal bar on the top of the cage. This allowed the forelimbs to have contact with the grid floor and allowed the animals to move around the cage for free access to food and water. The suspension height was adjusted to prevent the hindlimbs from touching any supporting surface while maintaining a suspension angle of ~30°. Animals were kept at 22 °C in a light-controlled environment (12:12 h light–dark cycle) with food and water ad libitum. All experiments were performed at the Institute of Biomedical Problems, RAS, Russia. The Committee on Bioethics of the Russian Academy of Sciences reviewed and approved all animal experiments for this study (protocol 583; 31 May 2021). Internationally accepted regulations in compliance with ARRIVE guidelines [13] and rules of biomedical ethics were followed during this study. In the 7-day experiment, 2,5-month-old male Wistar rats obtained from the institutional vivarium were divided into 4 experimental groups: sedentary control with placebo (C, n = 8), 7-day hindlimb-suspended group with placebo (HS, n = 8), sedentary control with metformin administration (gavage with metformin; 300 mg/kg of body weight per day; C+M, n = 8) and 7-day hindlimb-suspended group with metformin administration (gavage with metformin; 300 mg/kg of body weight per day, 7HS+M, n = 8). Previous studies showed that a dose of metformin of 300 mg/kg body weight is sufficient to activate AMPK in animal tissue [48,55,74]. Metformin was dissolved in water in a concentration of 15 mg/mL. Rats received either water or metformin solution (~2 ml per rat) via gavage twice per day. Animal weights and soleus muscle weights are shown in Table 1.

We conducted an additional preliminary experiment to detect the levels of ATP and p-AMPK protein level in soleus muscle after 3 days of rat hindlimb suspension.

In the 3-day experiment, male 2,5-month-old Wistar rats were divided into 4 experimental groups: sedentary control with placebo (3C, n = 8), 3-day hindlimb-suspended group with placebo (3HS, n = 8), sedentary control with metformin administration (3C+M, n = 8) and a 3-day hindlimb-suspended group with metformin administration (3HS+M, n = 8). The dosage and protocol were equivalent to the previously described 7-day experiment.

Upon completion of the experiments, the rats were anesthetized via an intraperitoneal injection of 10% avertin solution at 5 ml/kg of body weight (Sigma-Aldrich Corp., St. Louis, MO, USA) and euthanized via neck dislocation. Soleus muscles of the experimental animals were dissected, weighed and frozen in liquid nitrogen for further analyses. 

### 4.2. ATP Content Evaluation

The ATP Colorimetric/Fluorometric Assay Kit (MAK190; Sigma, St. Louis, MO, USA) was used for measurements of the ATP content in soleus muscle samples according to the manufacturer’s instructions, as has been described previously [16]. 

ATP concentration was evaluated using the following formula:

ATP concentration = B ∗ DDF/V

B—amount of ATP in the sample well calculated based on the standard curve;

V—sample volume added into the wells (50 µL).

DDF (deproteinization dilution factor) was calculated using the following formula:

DDF = (500 µL + volume KOH (µL))/initial sample volume PCA.

### 4.3. Immunohistochemistry

The transverse frozen 8 μm thick sections of the soleus muscle samples were prepared with a Leica CM 1900 cryostat at 20 °C, dried at room temperature for 15 min, and incubated in PBST for 20 min. The immunostaining was performed as described previously [13]. Primary antibodies used in this study were MyHC I(β) slow, 1:100 (Sigma, USA) and MyHC fast, 1:60 (DSMZ, Germany) for overnight conditions at+4 Co. The anti-MyHC fast antibody used in this study does not distinguish between different fast MyHC isoforms. The secondary antibodies used were Alexa Fluor 546 (1:1000) and Alexa Fluor 488 (1:1000) (Waltham, Massachusetts, USA). The sections were examined and photographed using a Leica Q500MC fluorescence microscope with an integrated digital camera (TCM 300F, Leica, Braunschweig, Germany) at 20× objective magnification. The analysis of fast-type and slow-type CSA areas was performed using ImageJ 1.52a software. At least 10 cross-sections per sample were analyzed to determine the percentage of different muscle fiber types in the sample (n = 8) and at least 100 fibers of each type were evaluated in every sample. This number is sufficient to evaluate myofiber characteristics [75]. Fast myosin-negative myofibers were counted as slow-type fibers, while slow myosin-negative myofibers were accounted as fast-type fibers. The sum of slow-type and fast-type myofiber percentages may exceed 100% due to the presence of hybrid fibers. 

### 4.4. Protein Extraction

Frozen soleus muscles were used for protein extraction. Muscle samples from each rat were sectioned with a Leica cryostat (20 μm, 10–15 mg) in the mid-belly region and homogenized for 25 min in RIPA buffer (#sc-24948, Santa Cruz Biotechnology, Dallas, TX, USA) using TissueLyser LT (QIAGEN, Hilden, Germany). Complete Protease Inhibitor Cocktail (#sc-29130, Santa Cruz Biotechnology, Dallas, TX, USA), Phosphatase Inhibitor Cocktail B (#sc-45045, Santa Cruz Biotechnology, Dallas, TX, USA), leupeptin (10 µg/mL), aprotinin (10 μg/mL), and pepstatin A (10 µg/mL) were added to the RIPA buffer prior to homogenization. Samples were centrifugated for 15 min at 2000× *g* and supernatant was collected and stored at −85 °C. 

Nuclear extracts were prepared from 50 mg of soleus muscle using NE-PER nuclear and cytoplasmic extraction reagents (Thermo Scientific, USA). Complete Protease Inhibitor Cocktail (Santa-Cruz, Dallas, Texas, USA), Phosphatase Inhibitor Cocktail B (Santa Cruz), PMSF (1 mM), aprotinin (10 μg/mL), leupeptin (10 μg/mL), and pepstatin A (10 μg/mL) were used to maintain extract integrity and function. Nuclear extracts were dialyzed by means of Amicon Ultra-0.5 centrifuge filters (EMD Millipore, Burlington, Massachusetts, USA).

The Bradford Protein Assay (Bio-Rad Laboratories, Hercules, CA, USA) and an Epoch spectrophotometer (Bio-Tek Instruments, Winooski, Vermont, USA) were used to determine protein concentration in each sample. The supernatant fluid was diluted with 2X sample buffer (5.4 mM Tris-HCl, pH 6.8, 4% SDS, 20% glycerol, 10% β-mercaptoethanol, 0.02% bromphenol blue) and stored at -85 °C for immunoblot procedures.

### 4.5. Western Blot Analysis

After dilution in sample buffer, the samples were run on 10% SDS-PAGE (20 µg/lane) using mini-Protean 3 Cell (Bio-Rad Laboratories, Hercules, USA). Proteins were transferred to a nitrocellulose membrane (Bio-Rad Laboratories, Hercules, CA, USA) for two hours. After the transfer, the membranes were stained with 0.3% of Ponceau S (Sigma, Saint Louis, USA) solution dissolved in 5% acetic acid and washed several times in PBS/0.1% Tween-20. Membranes were blocked with blocking buffer (5% nonfat milk powder, PBS pH 7.4, and 0.1% Tween-20) and incubated overnight at 4 °C with the solution of primary antibodies. 

The following primary antibodies were used: p-AMPK (1:1000, #2535), AMPK (1:1000, #5831), p-ACC (1:1000, #11818), ACC (1:1000, #3676), p-CaMKII (1:1000, #12716), and CaMKII (1:1000, #3362), all from Cell Signaling Technology, Danvers, Massachusetts, USA; HAT P300 (1:500, USA, # ab231010) and NFATc1 (1:1000, # ab2796), from Abcam, USA, and MEF2-D (1:1000, EMD Millipore, USA, # AB2263)-phosphorylated Tyr 180/Thr182MAP kinase p38 (1:500, GeneTex, Inc., USA, # GTX59567) and total MAP kinase p38 (1:500,Cell Signaling Technology, USA, #9212). Antibody against GAPDH (1:10000, G 041; ABM, Richmond, Canada) was used for normalization in the total protein fraction and lamin B1 antibody (1:500, # ab16048) was used for normalization in the nuclear protein fraction. 

Following three washes with PBS-Tween (PBS and 0.1% Tween-20), the membranes were incubated for one hour at room temperature with secondary antibodies (horseradish peroxidase-conjugated goat anti-rabbit (1:30000, #111-035-003, Jackson Immuno Research, West Grove, PA, USA) or goat anti-mouse (1:20000, #1706516, Bio-Rad, Hercules, CA, USA)). For all the blots, a negative control analysis for secondary antibody specificity was performed. Then, the membranes were washed in PBST three times and incubated with Clarity Western ECL Substrate (Bio-Rad Laboratories, Hercules, CA, USA). A C-DiGit Blot Scanner (LI-COR Biotechnology, Lincoln, NE, USA) was used for blot evaluation and Image Studio C-DiGit software was used for the analysis. For all blots, equal protein loading was verified via membrane staining with Ponceau S. The protein expression data for each group are expressed as a percentage of the control group values.

### 4.6. RNA Isolation and Reverse Transcription

RNA isolation from frozen soleus muscle was performed using the RNeasy Micro Kit (Qiagen, Hilden, Germany) according to the recommendations of the manufacturer. Microspin FV-2400 (Biosan, Riga, Latvia) was used for homogenization; after that samples were centrifugated at room temperature for 3 min at 10,000× *g*. The concentration of RNA in the samples was evaluated using a NanoPhotometer (Implen GmbH, Munich, Germany). Reverse transcription was performed by incubating 0.5 micrograms of RNA, random hexamers d(N)6, dNTPs, RNase inhibitor, and MMLV (Moloney Murine Leukemia Virus) reverse transcriptase (Syntol, Moscow, Russia) for 60 min at 42 °C.

### 4.7. Quantitative PCR Analysis

Amplification was performed using Quantitect SYBR Green Master Mix (Syntol, Moscow, Russia) and 10 pM of each forward and reverse primer. Sequences of the primers are presented in Table 2. Primers were synthesized by Syntol (Moscow, Russia). Annealing was performed at temperature optimal for each PCR primer-pair. iQ5 Multicolor Real-Time PCR Detection System (Bio-Rad Laboratories, Hercules, CA, USA) was used for amplification. Melting curve analysis was used to verify the specificity of the primers. Relative quantification was performed with the Pfaffl method [76]. RPL19 was chosen for the normalization of all quantitative PCR analysis experiments in the current study. RPL19 mRNA expression did not differ among the experimental groups.

### 4.8. Statistical Analysis

All data are expressed as the median and interquartile range (0.25–0.75) of eight animals, or are otherwise indicated in the figure legend. Significant differences between groups were statistically analyzed using two-way ANOVA followed by Tukey’s test. When the data did not meet normality criteria, data were analyzed by nonparametric methods (Kruskal–Wallis test followed by Dunnett’s test). Differences with values of *p* ≤ 0.05 were considered statistically significant.

## Figures and Tables

**Table 1:** Body weights and m soleus weights of the experimental animals. *—significant differences of C group. &—significant differences of C+M group (*p* < 0.05).

Group	Animal Weight, g	*M Soleus* Weight, mg
C	208.7 ± 33	91.2 ± 17
C+M	202.8 ± 30	93.2 ± 16
7HS	187.4 ± 30	56.1 ± 7 *&
7HS+M	174.6 ± 24 *&	52.7 ± 10 *&

**Table 2:** PCR primers used in the study.

Gene Description	*Primer Sequence*
Myh7 (MyHC I(β))	5′-ACAGAGGAAGACAGGAAGAACCTAC-3′ 5′-GGGCTTCACAGGCATCCTTAG-3′
Myh2 (MyHC IIa)	5′-TATCCTCAGGCTTCAAGATTTG-3′ 5′-TAAATAGAATCACATGGGGACA-3′
Myh4 (MyHC IIb)	5′-CTGAGGAACAATCCAACGTC-3′ 5′-TTGTGTGATTTCTTCTGTCACCT-3′
Myh1 (MyHC IId/x)	5′-CGCGAGGTTCACACCAAA-3′ 5′-TCCCAAAGTCGTAAGTACAAAATGG-3′
SERCA1	5′-GACTGAGTTTGGGGAACAGCT-3′ 5′-GAGGTGGTGATGACAGCAGG-3′
SERCA2	5′-GAAGCAGTTCATCCGCTACCTCA-3′ 5′-GCAGACCATCCGTCACCAGA-3′
PGC1alpha	5′-GTGCAGCCAAGACTCTGTATGG-3′ 5′-GTCCAGGTCATTCACATCAAGTTC-3′
SOX6	5′-TCAAAGGCGATTTACCAGTGAC-3′ 5′-TTGTTGTGCATTATGGGGTGC-3′
Rcan1 (MCIP1.4)	5′-CCGTTGGCTGGAAACAAG-3′ 5′-GGTCACTCTCACACACGTGG-3′
RPL19	5′-GTACCCTTCCTCTTCCCTATGC-3′ 5′-CAATGCCAACTCTCGTCAACAG-3′

## Data Availability

The data that support the findings of the study are available from the corresponding author upon reasonable request.
